# An atypical case of neurosarcoidosis presenting with neovascular glaucoma

**DOI:** 10.1186/s12348-018-0149-4

**Published:** 2018-04-18

**Authors:** Melissa Vereecken, Karolien Hollanders, Deborah De Bruyn, Virginie Ninclaus, Julie De Zaeytijd, Ilse De Schryver

**Affiliations:** Department of Ophthalmology, University Hospital Ghent, De Pintelaan 185, 9000 Ghent, Belgium

**Keywords:** Neurosarcoidosis, Neuro-ophthalmic sarcoidosis, Compressive ischemic optic neuropathy, Neovascular glaucoma, Optic nerve mass granuloma

## Abstract

**Background:**

Sarcoidosis, a multisystem, granulomatous disorder, sometimes manifests with a neuro-ophthalmic subtype. The latter can pose a diagnostic challenge, especially when ocular symptoms appear before systemic involvement, as the clinical picture then can be non-specific and systemic laboratory and standard imaging investigations can be negative.

**Findings:**

A 71-year-old woman presented with a 4-month history of sudden-onset visual loss in the left eye. Slit lamp examination revealed anterior chamber cells, iris, and angle neovascularization. Fundoscopy showed a pale edematous optic nerve head surrounded with intraretinal hemorrhages and yellow retinal infiltrates. The vasculature was very narrow to absent. Indeed, fluorescein angiography filling was limited to the (juxta-)papillary region. An extensive systemic work-up revealed a monoclonal gammopathy and absence of any inflammatory markers. On MRI, a mass infiltration of the intraorbital and the intracranial optic nerve was visible. Additional PET-CT scan revealed hilar lymph nodes. A transbronchial biopsy demonstrating a non-caseating granulomatous lesion led to the diagnosis of sarcoidosis and thus neurosarcoidosis. Treatment with high-dose prednisone and azathioprine was started to avoid progression and subsequent visual loss in the other eye.

**Conclusions:**

A patient with neurosarcoidosis presenting with compressive ischemic optic disc edema and neovascular glaucoma is described, increasing the diversity of clinical presentations and confirming the diagnostic challenge of neurosarcoidosis.

## Introduction

Sarcoidosis is a multisystem, granulomatous, inflammatory disorder with both systemic and ocular manifestations. Systemically, the lungs are most frequently involved, although also the liver, eyes, lymph nodes, skin, and central nervous system (CNS) can be affected [17, 30]. Because of the possible non-simultaneous disease in all those systems, sarcoidosis is known as the great mimicker and can exhibit symptoms of neoplastic, infectious, and inflammatory disorders. Ophthalmologists are mainly familiar with primary ocular involvement occurring in approximately 25–60% of patients, with uveitis being the most frequent manifestation [14]. On the other hand, in patients with CNS involvement, neurosarcoidosis (5–15%), a minority (1–5%) is faced with problems related to the optic nerve, chiasm, and/or visual tract known as neuro-ophthalmic sarcoidosis [16]. A diagnosis of ophthalmic neurosarcoidosis (NS) often poses a real challenge, especially when these neuro-ophthalmic symptoms are present before systemic involvement. The main target then is to establish the presence of systemic sarcoidosis, facilitating the histological confirmation of the diagnosis. As such, an early diagnosis may avoid further significant morbidity.

## Case report

**Fig. 1:**
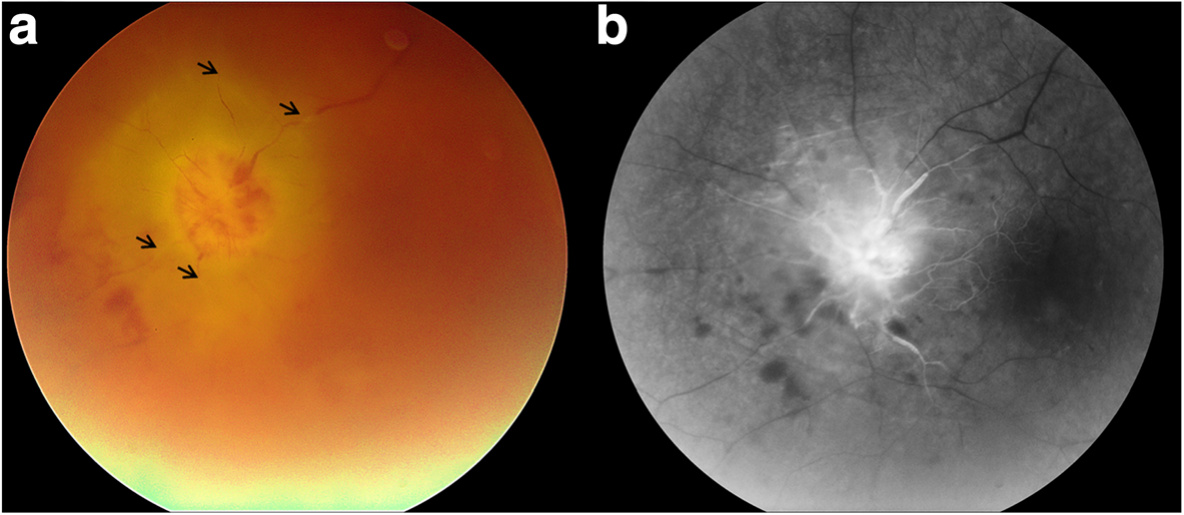
**a** Color photograph of the left disc and macula shows pale disc edema, marked irregularity of the retinal vasculature (arrows) and hemorrhages inferior and nasal to the optic disc. **b** The arteriovenous-phase fluorescein angiogram of the left disc and macula shows an optic disc staining, as well as extensive non-perfusion of the retinal vasculature and venous congestion. Of note, the blot hemorrhages inferior to the optic disc cause irregular hypofluorescent blockage

A 71-year-old Caucasian woman was referred because of a painless, rapidly progressive visual loss in the left eye (LE) within 1 week. Best corrected visual acuity was 6/6 in the right eye (RE) and no light perception in the LE. Examination of the RE was completely unremarkable. The left eye had an absolute afferent pupillary defect. Intraocular pressure was within normal limits. Slit lamp examination of the LE showed iris neovascularization, discrete angle neovascularization, and a few cells in the anterior chamber. Fundus examination revealed a pale optic disc edema surrounded with round intraretinal hemorrhages and round yellow retinal infiltrates. Few choroidal folds were visible inferonasal of the disc. The vasculature, both the arteries and veins, was very narrow to absent (Fig. 1a). On fluorescein angiography, filling was limited to the disc and juxta-papillary region until the late phase with leakage of the disc due to the disc edema. The macula and (mid-)periphery were non-perfused without any neovascularization (Fig. 1b). Unfortunately, there was a rapid evolution towards neovascular glaucoma (NVG) in need of treatment with pressure-lowering drops, panretinal photocoagulation, intravitreal injection with anti-vascular growth factor (anti-VEGF), and eventually ablation of the ciliary body.

In the differential diagnosis, ischemic, inflammatory, and neoplastic causes were withheld. An extensive systemic work-up was performed which included a full blood count, erythrocyte sedimentation rate (ESR), C-reactive protein (CRP), serology, autoantibodies (ANCA, ANF, anti-cardiolipin, lupus anticoagulans), protein electrophoresis, Mantoux test, cardiovascular work-up, chest radiography, and MRI of the orbit. Laboratory findings showed normal ESR and CRP and a known and stable monoclonal gammopathy of unknown significance (MGUS) (Table 1)*.* Additional laboratory tests, including angiotensin-converting enzyme (ACE), liver function, and hypercalcemia, were negative. Infectious serology as for tuberculosis, toxoplasmosis, syphilis, and borreliosis was also negative.

**Table 1:** Laboratory results

Laboratory results	
Serology	• Toxoplasmosis IgG positive and IgM negative • Epstein-Barr virus IgM and IgG negative • Cytomegalovirus IgG positive and IgM negative • *Bartonella henselae* IgG and IgM negative • Borrelia/lyme screening: 0.16 (> 1.1 = positive) • HIV Ag and Ab negative • Syphilis TPPA negative
Inflammation	• ESR negative 18 mm/h • CRP negative 2.4 mg/L
Electrophoresis	• Albumine 57.3% (53.7–66.0%) • Alpha 1-globulines 6.2% (4.8–8.4%) • Alpha 2-globulines 10.1% (6.4–12.5%) • Beta-globulines 9.2% (8.9–14.6%) • Gamma-globulines 17.2% (8.7–17.7%)
	ACE 38 U/L (19–92 U/L)
Rheumatology	• Anti-nuclear antibodies negative • Anti-neutrophil cytoplasmic antibody negative

**Fig. 2:**
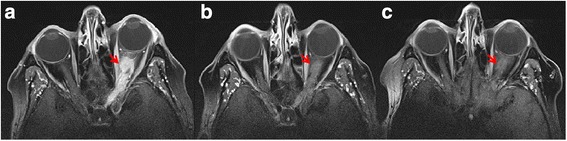
Orbital MRI shows a perineural mass of the left optic nerve (arrows). **a** A contrast-enhancing lesion compressing and infiltrating the left optic nerve is seen on the initial MRI scan. **b** A second fat suppression MRI scan shows a perineural T1 hypo-intense mass reaching from the bulbus oculi up to the optic chiasm, with homogeneous contrast enhancement. **c** Two months after the start with steroids, a distinctly smaller orbital mass is seen on fat suppression MRI scan with residual perineural thickening of the optic nerve without contrast enhancement

All the autoantibodies mentioned above remained negative, and other immunological disorders such as granulomatosis with polyangiitis and systemic lupus erythematosus were ruled out. Chest X-ray and cardiovascular investigation, including carotid duplex ultrasound, were normal. On the contrary, MRI orbit with fat suppression revealed a perineural T1 low signal intense mass reaching from the bulbus oculi up to the optic chiasm, with homogeneous contrast enhancement (Fig. 2a, b). The differential diagnosis included an optic nerve sheath meningioma, an optic glioma, a lymphoma, and/or an inflammatory mass. Further investigation with positron emission tomography (PET)-CT scan demonstrated mediastinal and bilateral hilar lymph nodes, aside from a hypermetabolic lesion around the optic nerve. Together with the known MGUS, the lymphadenopathy was highly suspicious of a lymphoma. However, a transbronchial biopsy to further differentiate the hilar lesion showed a non-caseating granuloma, making the diagnosis of a lymphoma less likely and pointing to the diagnosis of systemic sarcoidosis, and subsequently NS. Since, the mass around the optic nerve was therefore indirectly diagnosed as a sarcoid granuloma compressing the optic nerve and its vasculature causing disc edema, retinal ischemia, and eventually NVG. Once the diagnosis of NS was made, high dose per oral prednisone was commenced together with steroid-sparing azathioprine. The therapeutic response was confirmed through a repeat MRI of the orbit (Fig. 2c), showing a reduction of the optic nerve granuloma with persistent residual perineural thickening of the optic nerve, but without contrast enhancement.

## Discussion

Sarcoidosis is a chronic multisystem, inflammatory disease characterized by non-necrotizing granuloma affecting multiple organ systems [32]. Ocular disease may be the initial manifestation or develop at any stage of systemic sarcoidosis and can involve any part of the eye and its adnexal tissues [21] (Table 2). The most common ocular manifestation is uveitis [14].

**Table 2:** Diagnostic criteria for ocular sarcoidosis [14]

Proposed criteria for the diagnosis of ocular sarcoidosis	
Definite	• Biopsy-proved diagnosis • Compatible uveitis
Presumed	• No biopsy • Positive chest X-ray for sarcoidosis with hilar lymphadenopathy • Compatible uveitis
Probable	• No biopsy • Negative chest X-ray • 3 suggestive intraocular signs* • 2 positive investigational tests**
Possible	• Negative biopsy • 4 suggestive intraocular signs* • 2 positive investigational tests**

*Mutton-fat keratic precipitates and/or iris nodules (Koeppe/Bussacca)–trabecular meshwork nodules and/or tent-shaped peripheral anterior synechiae–snowballs–multiple chorioretinal peripheral lesions–peri-phlebitis and/or macroaneurisms–optic disc nodule/granuloma/solitary choroidal nodule—bilaterality

**Negative tuberculin test–elevated ACE and/or elevated serum lysozyme–\abnormal liver enzyme tests–chest CT

Involvement of the CNS, referred to as neurosarcoidosis (NS), occurs in only 5–15% of cases [14, 22] with cranial neuropathies as the most frequent neurological manifestation [12, 23]. Neuro-ophthalmic sarcoidosis is a specific subset of NS of unknown frequency that involves the afferent and efferent visual systems [11]. NS however can cause secondary pathology in the eye [5, 7, 9, 29]. Optic nerve head granuloma can be the first presentation of NS [15].

The diagnosis of systemic sarcoidosis requires suggestive clinical findings, histologic demonstration of non-caseating granulomas and exclusion of other diseases. When the optic nerve is involved, there is difficulty in making a tissue diagnosis. Validated diagnostic criteria for NS are not available. However, [34] proposed diagnostic criteria for NS (Table 3), which can be definite, probable, or possible [34]. The International Workshop on Ocular Sarcoidosis (IWOS) published more recent criteria in 2009 [14]. Both sets of criteria have limitations in our case. According to Zajicek et al., a diagnosis of probable NS was made in our patient. A definite diagnosis needs a biopsy of the CNS. If we look at the IWOS criteria, the place of biopsy is different. To meet a definite diagnosis of ocular sarcoidosis (IWOS), a positive biopsy of the lungs and a compatible uveitis are needed. A CNS biopsy is not necessary.

**Table 3:** Diagnostic criteria for neurosarcoidosis [34]

Proposed criteria for the diagnosis of neurosarcoidosis	
Definite	• Positive nervous system histology • Clinical presentation suggestive of neurosarcoidosis
Probable	• Clinical syndrome suggestive of neurosarcoidosis • Laboratory support for central nervous system inflammation* • Evidence for systemic sarcoidosis** • Exclusion of alternative diagnosis
Possible	• Clinical presentation suggestive of neurosarcoidosis • Exclusion of alternative diagnoses where the above criteria are not met

*Elevated levels of cerebrospinal fluid protein and/or cells—the presence of oligoclonal bands and/or MRI evidence compatible with neurosarcoidosis

**Positive histology—Kveim test—at least two indirect indicators from gallium scan, chest imaging, and serum ACE

Nevertheless, according to the IWOS criteria, the classification of this case is more difficult. The ocular findings alone do not meet the terms because the patient only has one ocular sign (optic disc granuloma) instead of three. However, the additional two necessary laboratory tests are positive (chest CT scan and lung biopsy) and confirm the diagnosis. If we give more value to the positive biopsy than to a compatible uveitis, then we can speak of a definite sarcoidosis. However, without this biopsy, the patient does not meet the IWOS classification.

This case is not the only one that does not meet the IWOS criteria. Acharya et al. validated the IWOS diagnostic criteria and estimated the sensitivity and specificity [1]. They included 884 uveitis patients. Most clinical signs were only 37–50% sensitive. The IWOS criteria were not sufficient to classify all patients with sarcoid uveitis. Even in patients with biopsy-proven sarcoidosis, 10% showed no clinical signs or only had bilaterality. Approximately 40% of ocular sarcoidosis suspected cases did not meet any of the IWOS classes. This raises the question whether another classification system is warranted. Recent studies suggest that absolute lymphocyte count and serum concentrations of interleukin 2 receptor could be diagnostically helpful [1].

Our diagnostic evaluation included not only testing to confirm a clinical suspicion of NS but also examinations to exclude other etiologic diseases. Indeed, serology excluded infectious disorders. Laboratory testing was focused on finding signs of systemic sarcoidosis; however, inflammatory markers (ESR/CRP), ACE, liver function, and calcium metabolism were normal. However, the utility of ACE in NS is controversial, in which the predictive value of increased serum ACE levels is not optimal [2]. Moreover, there is still a lot of debate concerning the diagnostic value of ACE levels in cerebrospinal fluid (CSF) [10]. Additionally, other CSF abnormalities, such as elevated protein levels, lymphocytosis, pleocytosis, and hypoglycorrhachia, may also suggest the diagnosis of NS [26, 29]. However, all these tests are insensitive and non-specific for the diagnosis of NS [19]. In addition, a normal CSF study does not exclude NS [28]. Chest imaging on the other hand usually does demonstrate abnormalities in cases of suspected NS and strongly supports the diagnosis of NS if positive [8, 25, 26]. Therefore, a lumbar puncture was not performed in our patient.

Sarcoidosis is known to produce various antibodies and can occur together with systemic lupus erythematosus [33]. Detection of autoantibodies was negative, ruling out other or additional immunological disorders.

Neuro-imaging was focused on finding or excluding a compressive lesion. MRI of the orbit showed a perineural mass with low signal intensity, extending from the bulbus oculi to the optic chiasm, including nerve sheath meningioma, glioma, lymphoma, and an inflammatory lesion in the differential diagnosis. Because brain MRI is sensitive for showing mass infiltration of the optic nerve but has poor specificity, additional imaging was performed [20, 27]. Subsequent body PET-CT scan revealed a hypermetabolic lesion from the bulbus oculi up to the chiasma, mediastinal lymph nodes, and bilateral hilar lymphadenopathy.

This image together with the presence of MGUS was suggestive for lymphoma. Indeed, literature has shown that MGUS can evolve into a lymphoma [13]. Initial imaging was therefore focused on detecting orbital lymphoma with compression of the optic nerve. Tissue biopsy is always essential for the final diagnosis, and PET-CT scan was thereby helpful to pinpoint sites for biopsy. Eventually, bronchoscopy with transbronchial biopsy established the diagnosis of sarcoidosis, which made the plausible diagnosis of lymphoma slim. Nevertheless, an association of sarcoidosis with lymphoproliferative malignancies has been reported [18]. Indeed, sarcoidosis can exhibit a polyclonal but not a monoclonal gammopathy [18]. Thus, once a diagnosis of probable NS is confirmed, one should always keep an open mind and undertake careful follow-up as in the absence of histological proof the possibility of two different diseases is not excluded.

Keeping in mind that bilateral optic nerve involvement [25, 26] is not unusual, treatment was started to protect the RE, although the left eye was functionally lost. Currently, evidence-based therapeutic guidelines are missing because of the rarity of NS. However, Frohman [11] published a NS treatment strategy, similar to the approach of Baughman et al. [3, 4]. In brief, high-dose steroids are the first line therapy, either intravenous or oral. If steroids fail, a steroid-sparing immunosuppressive such as mycophenolate mofetil, azathioprine, or methotrexate may be added. In refractory cases, anti-tumor necrosis factor agent can be used as a potential third-line therapeutic option. The choice of a second (and third) agent depends on the clinical and radiographical evolution and safety profile [11]. The patient responded well to high-dose steroids, but immunosuppressive treatment with azathioprine was added in order to limit steroid-induced complications and protect the fellow eye. Cerebral imaging was repeated in order to monitor the treatment.

Dubbed the great mimicker, NS has a great variety of presentations. It can occur at all ages, and all races, being relatively more common in Caucasian patients of Northern European descent. The diagnosis should therefore always be considered in these individuals. The combination of clinical signs presented here, optic neuropathy, widespread retinal ischemia, and NVG without a history of intraocular inflammation, did not provoke immediate suspicion of NS. The sarcoid granuloma compressed the optic nerve and the vasculature resulting in an occlusion of the retinal circulation and secondary extensive retinal ischemia, demonstrated by fluorescein angiography. Retinal ischemia produces angiogenic factors, and NVG arises in response [6]. Indeed, increased VEGF levels in the aqueous of patients with NVG have been demonstrated [31]. Interestingly, in patients with sarcoidosis, transcription of angiogenic cytokines such as VEGF is known to be generally upregulated [24]. If this might have accelerated, evolution towards NVG in our patient is unclear.

## Conclusions

A patient with neurosarcoidosis presenting with compressive ischemic optic disc edema, widespread retinal ischemia, and neovascular glaucoma is described, increasing the diversity of clinical presentations and confirming the diagnostic challenge of neurosarcoidosis. The diagnosis should be kept in mind, even if the clinical signs do not make the complete diagnostic criteria, according to current criteria. We propose that sarcoidosis should still be considered if a patient presents with only one clinical sign, particularly if the patient is of an at-risk demographic. It stresses the importance so appropriate therapy can be timely initiated to avoid further organic loss.

## Abbreviations

ACE: Angiotensin-converting enzyme
CNS: Central nervous system
CRP: C-reactive protein
CSF: Cerebrospinal fluid
CT: Computed tomography
ESR: Erythrocyte sedimentation rate
IWOS: International Workshop on Ocular Sarcoidosis
KP: Keratic precipitates
LE: Left eye
MGUS: Monoclonal gammopathy of unknown significance
MRI: Magnetic resonance imaging
MS: Multiple sclerosis
NS: Neurosarcoidosis
NVG: Neovascular glaucoma
PET: Positron emission tomography
RE: Right eye
VEGF: Vascular endothelial growth factor

## References

[1] Acharya NR, Browne EN (2018). Distinguishing features of ocular sarcoidosis in an international cohort of uveitis patients. Ophthalmology.

[2] Baarsma GS, La Hey E (1987). The predictive value of serum angiotensin converting enzyme and lysozyme levels in the diagnosis of ocular sarcoidosis. Am J Ophthalmol.

[3] Baughman RP, Lower EE (2012). Management of ocular sarcoidosis. Sarcoidosis Vasc Diffuse Lung Dis.

[4] Baughman RP, Nunes H (2012). Therapy for sarcoidosis: evidence-based recommendations. Expert Rev Clin Immunol.

[5] Brindeau C, Glacet-Bernard A (1999). Sarcoid optic neuropathy. Journal francais d'ophtalmologie.

[6] Brown GC, Magargal LE (1984). Neovascular glaucoma. Etiologic considerations. Ophthalmology.

[7] Caplan L, Corbett J (1983). Neuro-ophthalmologic signs in the angiitic form of neurosarcoidosis. Neurology.

[8] Chapelon C, Ziza JM (1990). Neurosarcoidosis: signs, course and treatment in 35 confirmed cases. Medicine.

[9] Constantino T, Digre K (2000). Neuro-ophthalmic complications of sarcoidosis. Semin Neurol.

[10] Dale JC, O'Brien JF (1999). Determination of angiotensin-converting enzyme levels in cerebrospinal fluid is not a useful test for the diagnosis of neurosarcoidosis. Mayo Clin Proc.

[11] Frohman LP (2015). Treatment of neuro-ophthalmic sarcoidosis. J Neuroophthalmol.

[12] Frohman LP, Grigorian R (2001). Neuro-ophthalmic manifestations of sarcoidosis: clinical spectrum, evaluation, and management. J Neuroophthalmol.

[13] Glavey SV, Leung N (2016). Monoclonal gammopathy: the good, the bad and the ugly. Blood Rev.

[14] Herbort CP, Rao NA (2009). International criteria for the diagnosis of ocular sarcoidosis: results of the first International Workshop On Ocular Sarcoidosis (IWOS). Ocul Immunol Inflamm.

[15] Hickman SJ, Quhill F (2016). The evolution of an optic nerve head granuloma due to sarcoidosis. Neuro-Ophthalmology.

[16] Hoitsma E, Faber CG (2004). Neurosarcoidosis: a clinical dilemma. Lancet Neurol.

[17] Hosoda Y, Yamaguchi M (1997). Global epidemiology of sarcoidosis. What story do prevalence and incidence tell us?. Clin Chest Med.

[18] Hunninghake GW, Crystal RG (1981). Mechanisms of hypergammaglobulinemia in pulmonary sarcoidosis. Site of increased antibody production and role of T lymphocytes. J Clin Invest.

[19] Khoury J, Wellik KE (2009). Cerebrospinal fluid angiotensin-converting enzyme for diagnosis of central nervous system sarcoidosis. Neurologist.

[20] Mana J (2002). Magnetic resonance imaging and nuclear imaging in sarcoidosis. Curr Opin Pulm Med.

[21] Menezo V, Lobo A (2009). Ocular features in neurosarcoidosis. Ocul Immunol Inflamm.

[22] Mercan M, Akyol A (2015). A case of sarcoidosis of the central nervous system and Orbita. Case Rep Med.

[23] Mijajlovic M, Mirkovic M (2014). Neurosarcoidosis: two case reports with multiple cranial nerve involvement and review of the literature. Biomed Pap Med Fac Univ Palacky Olomouc Czech Repub.

[24] Mikami R, Sekiguchi M (1986). Changes in the peripheral vasculature of various organs in patients with sarcoidosis—possible role of microangiopathy. Heart Vessel.

[25] Nozaki K, Judson MA (2012). Neurosarcoidosis: clinical manifestations, diagnosis and treatment. Presse Med.

[26] Pawate S, Moses H (2009). Presentations and outcomes of neurosarcoidosis: a study of 54 cases. QJM.

[27] Pickuth D, Heywang-Kobrunner SH (2000). Neurosarcoidosis: evaluation with MRI. J Neuroradiol.

[28] Sharma OP (1997). Neurosarcoidosis: a personal perspective based on the study of 37 patients. Chest.

[29] Stern BJ, Krumholz A (1985). Sarcoidosis and its neurological manifestations. Arch Neurol.

[30] Tozman EC (1991). Sarcoidosis: clinical manifestations, epidemiology, therapy, and pathophysiology. Curr Opin Rheumatol.

[31] Tripathi RC, Li J (1998). Increased level of vascular endothelial growth factor in aqueous humor of patients with neovascular glaucoma. Ophthalmology.

[32] Valeyre D, Prasse A (2014). Sarcoidosis. Lancet.

[33] Weinberg I, Vasiliev L (2000). Anti-dsDNA antibodies in sarcoidosis. Semin Arthritis Rheum.

[34] Zajicek JP, Scolding NJ (1999). Central nervous system sarcoidosis—diagnosis and management. QJM.

